# Micro- and Nanotechnologies for Optical Neural Interfaces

**DOI:** 10.3389/fnins.2016.00070

**Published:** 2016-03-08

**Authors:** Ferruccio Pisanello, Leonardo Sileo, Massimo De Vittorio

**Affiliations:** ^1^Center for Biomolecular Nanotechnologies, Istituto Italiano di TecnologiaLecce, Italy; ^2^Dipartimento di Ingegneria dell'Innovazione, Università del SalentoLecce, Italy

**Keywords:** nanotechnology, optogenetics, nanoparticles, neural interfaces, optical fibers

## Abstract

In last decade, the possibility to optically interface with the mammalian brain *in vivo* has allowed unprecedented investigation of functional connectivity of neural circuitry. Together with new genetic and molecular techniques to optically trigger and monitor neural activity, a new generation of optical neural interfaces is being developed, mainly thanks to the exploitation of both bottom-up and top-down nanofabrication approaches. This review highlights the role of nanotechnologies for optical neural interfaces, with particular emphasis on new devices and methodologies for optogenetic control of neural activity and unconventional methods for detection and triggering of action potentials using optically-active colloidal nanoparticles.

## Introduction

The activity and interconnections of the billions of neurons in the human brain determine the function of our senses, dictate our motor choices, form memories, and guide behavior. Understanding, monitoring and manipulating neural activity with high spatial and temporal resolution *in vivo* and on a large number of neurons is mandatory for a deeper knowledge of neural circuitry, and to shine light on causal relations between neurons or between neurons and behavior.

New strategies and technologies to systematically monitor thousands of functional links that each neuron forms with other neurons are being developed. Nanoscience and nanotechnology can play a key role in developing new ideas and experimental approaches to create detailed maps of the human and mammalian brain (Nanotechnology and Neuroscience, 2014). Among new approaches for neuroscience, optical methods are very promising for both recording and manipulating neural activity. A major breakthrough in this respect has been the advent of optogenetics (Boyden et al., 2005), relying on the genetic expression of exogenous light-gated ion channels and ion pumps to control neuronal activity, allowing unprecedented causal manipulation of specific neural circuits.

In optogenetic experiments light of visible wavelengths is shined onto single neurons or, feasibly, large brain regions, to activate or inhibit specific classes of neurons, while simultaneously recording electrophysiological data or monitoring behavior in freely moving animals. However, due to induced tissue damage and light scattering and absorption, light delivery *in vivo* and in deep brain regions of animal models is still very challenging, and far from being effective (Stujenske et al., 2015). Both acute and chronic optical implants need to meet several requirements for different experiments: site specific light delivery or uniform illumination of large brain volumes, low physical, and thermal tissue damage, biocompatibility, high fidelity and minimized photoelectric artifacts, high switching speed, and tunable wavelength (Pisanello M. et al., 2014; Warden et al., 2014; Grosenick et al., 2015). Additionally, optical methods can be exploited for simultaneous light collection for all-optical manipulation and monitoring of neural activity, by using light-based genetically encoded neural activity indicators (GEAIs), such as fluorescent Ca^2+^ indicators or voltage sensitive dyes (VSD; Cui et al., 2013, 2014; Gunaydin et al., 2014).

This review encompasses the latest developments and technologies in the field of light delivery and possible approaches for optical monitoring of neuronal activity *in vivo*. A variety of different nanotechnologies and optical methods applied to neuroscience are presented, including active implanted LEDs, passive arrayed waveguides, nanomachined tapered fibers, and self-organized colloidal nanostructures.

## Top-down fabrication processes for multipoint optogenetic stimulation

Optogenetics—“the combination of optics and genetics to achieve gain or loss of functions of well-defined cellular events in specific cells of living tissue” (Deisseroth, 2011)– is widely adopted in the central nervous system on animal models to modulate neural activity and to regulate release of specific neurotransmitters (Adamantidis et al., 2007; Aravanis et al., 2007; Petreanu et al., 2009; Lin et al., 2013). This is achieved through the use of specific transmembrane proteins, called opsins, which respond to light by generating a flow of ions across the cellular membrane, acting as light-gated ion channels. An example of opsin used to trigger action potentials is Channelrhodopsin 2 (ChR2), a non-specific cation channel used to depolarize the neuron (Nagel et al., 2003). Inhibition of neural activity can instead be achieved by using Halorhodopsin (Halo) and Archaerhodopsins (Arch), light-driven ion-pumps used to hyperpolarize the cell, therefore inhibiting the generation of action potentials by reducing the probability of supra-threshold events (Nagel et al., 2003; Fenno et al., 2011; Tye and Deisseroth, 2012). ChR2, Halo and Arch, as well as many other membrane proteins, can be delivered into the brain by means of transfection approaches such as in utero electroporation, viral transfection or transgenic crossing, all allowing for gene delivery only to molecularly-defined classes of neurons (Han, 2012). This latter is the main advantage of optogenetics with respect to electrical stimulation of neural activity: light can be used to modulate electrical activity only of genetically-defined neural sub-populations without affecting nearby neurons of a different type, still allowing for post-synaptic effects to take place. After its first use in mammalian neurons in 2005 (Boyden et al., 2005), optogenetics is now adopted in several animal models from Caenorhabditilis elegans to primates and, in particular, in mice and rats to study functional connectivity of specific classes of neurons and to identify their particular role in neural diseases and disorders.

In this framework, a crucial aspect is represented by technologies to deliver light into the brain. The neural tissue is, indeed, a highly scattering medium and microscopy-based techniques are still restricted to the shallower layers of the cortex (Warden et al., 2014). Standard experimental protocols based on the implantation of a fiber stub with a flat-cleaved end are limited by the single illumination spot and the small volume excited at the fiber tip, since the delivered light power is strongly attenuated after a few hundreds of micrometers (Aravanis et al., 2007; Yizhar et al., 2011). To interact with deeper brain structures, new generations of implantable devices are being developed (Pisanello M. et al., 2014; Warden et al., 2014; Grosenick et al., 2015). In this context, top-down nanotechnology fabrication processes are allowing for unprecedented functionalities and integration processes, which resulted in a minimized damage to the brain tissue during implantation and, simultaneously, to the possibility of optically control a wider brain volume. As well, micro, and nanotechnologies have been exploited for realizing multifunctional devices, which recently overtook the classic concept of “optrode” (e.g., a device for simultaneous optical control and electrical monitoring of neural activity Grosenick et al., 2015) and can now integrate microfluidic systems for *in situ* drug delivery (Canales et al., 2015; Jeong et al., 2015a) or other devices such as temperature sensors or photodetectors (Kim et al., 2013).

### Implanted μLED

**Figure 1:**
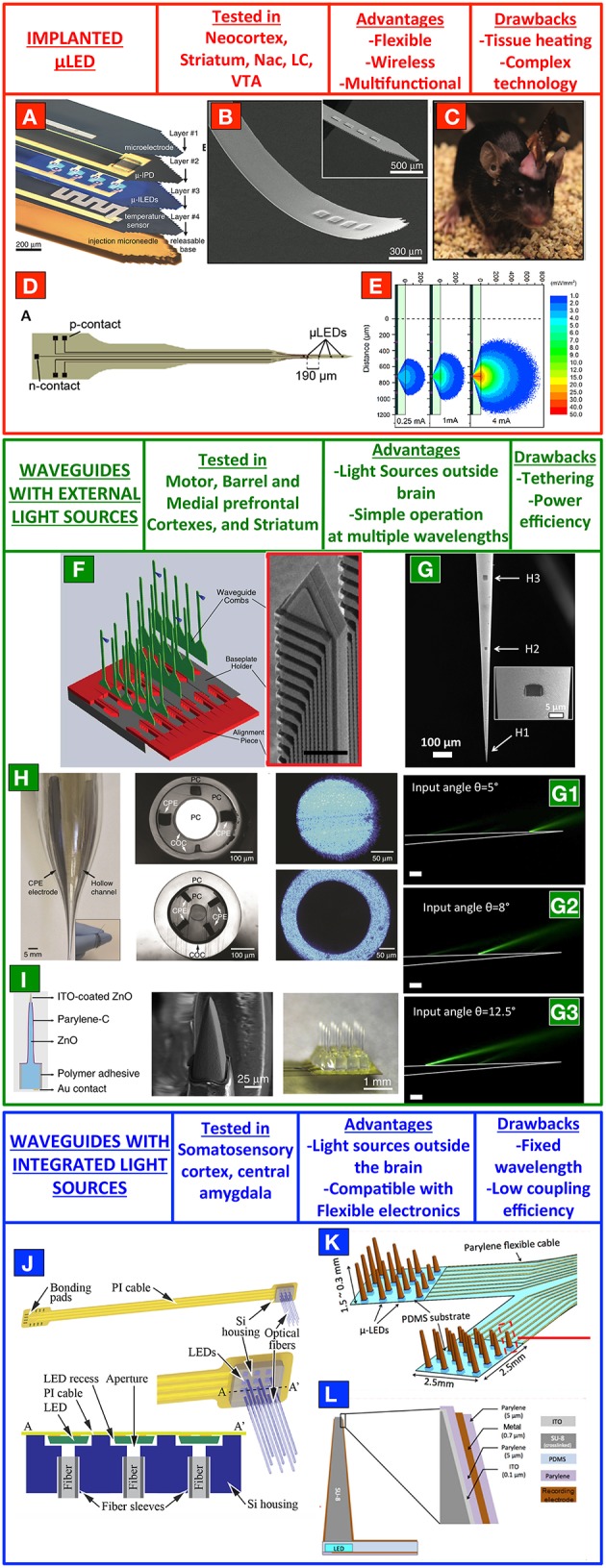
**Top-down fabrication processes for implantable multi-point stimulation devices. (A–C)** A wireless system consisting of μLEDs on a flexible shank. **(D)** A μLEDs device for site-selective stimulation of mouse neocortex. **(E)** Monte-Carlo simulations of the light radiation pattern from a single μLEDs implanted in the scattering tissue. **(F)** A 3D set of silicon oxynitride waveguides for custom optogenetic stimulations of defined points in a 3D fashion. **(G)** Multipoint-emitting optical fibers for stimulation of multiple brain regions with a single and tapered optical fiber. **(H)** Multifunctional polymeric fibers. **(I)** ZnO-based multipoint optical arrays for simultaneous optical control and electrical recording of neural activity. **(J)** Array of implantable optical fibers coupled to μLEDs on a flexible polyamide cable. **(K,L)** Array of tapered SU-8 waveguides coupled with μLEDs and electrodes for extracellular readout of neural activity. **(A–C)** are reproduced with permissions from Kim et al. (2013). Panels **(D,E)** are reproduced with permissions from McAlinden et al. (2015). Panel **(F)** is reproduced with permissions from Zorzos et al. (2012). Panels **(G–G3)** are modified from Pisanello F. et al. (2014). Panel **(H)** is reproduced with permissions from Canales et al. (2015). Panel **(I)** was reproduced with permissions from Lee et al. (2015). Panel **(J)** is reproduced with permission from Schwaerzle et al. (2015). Panels **(K,L)** are reproduced with permission from Kwon et al. (2015).

A very promising strategy to bring light to deep brain regions consists in using micro light emitting diodes (μLED) implanted directly in the target area. A straightforward and multi-purpose implementation of that was presented in 2013 in Kim et al. (2013) McCall et al. (2013). Kim et al. developed a method to realize releasable gallium nitride (GaN) μLEDs on a sapphire substrate (only 6.5 μm thick), which were then moved to thin plastic strips hosting multiple and independently addressable emitters. This was a constituent component of a layered implant that can be customized depending on the experimental needs, and can incorporate also other electrical elements such as platinum electrodes for extracellular recording or for electric stimulation, platinum temperature sensors, local heaters and microscale photodetectors (Figures 1A,B). The so-obtained stack is implanted via a releasable microneedle, extracted after the surgery. Driving electronics stays instead outside the skull and, interestingly, the system allows for straightforward wireless operation (Figure 1C) and, as very recently shown in Jeong et al. (2015a), for the integration of wireless-driven drug delivery systems. Blue light emitted by the μLEDs was used to stimulate dopaminergic neurons in the ventral tegmental area of untethered mice behaving in a complex environment containing sites for dopamine rewards, preferred by the ChR2-transfected animals during the experiment. This manuscript of Kim and co-workers represented a boost for related technologies, and in last 2 years other interesting approaches were suggested for μLED-based stimulation in other regions of the mouse brain. This is the case of the auditory pathway toward the brain (Hernandez et al., 2014). Hernandez et al. implanted a μLED to stimulate auditory brain stream responses via optogenetic excitation of spiral ganglion neurons in the mouse cochlea, showing that optical stimulation allows for a better frequency resolution with respect to classical monopolar electrical approach. Although this experiment was realized with a single μLED, the possibility to integrate multiple emitters on flexible shafts for multipoint stimulation has been recently demonstrated (Goßler et al., 2014). This technology exploits laser-lift-off to transfer from the original sapphire substrate the GaN μLEDs to polyamide films, having the proper mechanical properties to follow the cochlear curvature. Furthermore, multipoint stimulation with μLEDs on sapphire shanks was also demonstrated for site-selective stimulation of neocortical circuits, with Monte-Carlo simulations used to evaluate the broadening of emitted light induced by tissue scattering (McAlinden et al., 2015; Figures 1D,E).

Together with the advantages of a straightforward implementation on flexible devices, suitability for wireless operation and the possibility to integrate high-density emission points on a single shaft with a plurality of electrodes for extracellular recording, GaN-based μLED technology is still facing important challenges. In particular, the heat at the surface of the emitter poses an upper limit to the duration of light delivery stimuli. In McAlinden et al. (2013) this aspect was analyzed by a finite element model and Mc Allinden et al predicted that a conservative limit of 0.5°C increase of tissue temperature would not be reached until a pulse duration of ~200 ms (at 350 mW/mm^2^; McAlinden et al., 2013). By virtue of the multiple functionalities integrated in their flexible device, Kim et al. instead directly measured the temperature rise with a platimum sensor and measured temperature variations below 0.12°C for 10 ms-long pulses up to 20 Hz and power density up to ~15 mW/mm^2^ (Kim et al., 2013) The minimum distance between multiple emitters, instead, depends strongly on the presence of a dielectric material at the emitters/tissue interface and it is limited by tissue scattering and the Lambertian emission profile of the μLEDs (McAlinden et al., 2015).

### Waveguides-based implants

Another promising strategy to deliver light into the brain is represented by waveguides-based devices, which have recently seen the definition of important routes toward viable multi-point optogenetic stimulation. With respect to μLED-based approaches, they have the main advantages of keeping the light sources outside the tissue, thus avoiding direct heating induced by implanted electronics and to be able to change the delivered light wavelength based on experimental needs. In 2010 Zorzos et al. proposed a multipoint-emission device obtained by top-down fabrication process (Zorzos et al., 2010), with aluminum-coated multiple silicon oxynitride waveguides ending with corner mirrors to direct light laterally with respect to the implant direction. Each waveguide was coupled to external sources with single mode fibers, for overall outcoupling efficiency ranging from 23 to 33%. The same authors, in 2012, extended this technology to 3D arrays of independently addressable light emission points with multiple shanks arranged in three-dimensions using a micro-fabricated baseplate holder (Figure 1F; Zorzos et al., 2012). Each shank contains several light emission points in the same configuration of Zorzos et al. (2010), each of which can be independently addressed by external coupling systems such as digital micromirrors chips coupled to a microlenses array or galvanometric mirrors and a f-theta lens. In the same year, another approach was proposed by Abaya et al. based on a 3D array of sharpened waveguides allowing for stimulation at two different depths (Abaya et al., 2012). The SiO2 waveguides are realized through a dicing process, defining first the pyramidal shape of the shanks with a bevel blade and then the vertical pillars by deep kerfs. HF-based etching is then employed to thin the shanks and, finally, an annealing step is used to relieve internal stress and reduce the surface roughness.

Although these methods allow for a dynamic reconfiguration of the stimulation geometry during the experiment, a sever limit to viable *in vivo* implementation is represented by the pronounced implant cross section. A solution for that was proposed in 2014, exploiting the photonic properties of tapered optical fibers (Pisanello F. et al., 2014). The device is composed by a tapered optical fiber with a sub-micrometer tip diameter, with the tapered region covered with a gold layer to keep light confined into the waveguide. Light is allowed to outcouple into the brain through optical windows realized in the gold coating by Focused Ion Beam milling (Sileo et al., 2015), thus allowing optogenetic control of neural activity only at specific sites along the taper (Figure 1G). The active window can be selected by modifying the light coupling angle at the other end of the fiber, therefore injecting into the waveguide different subsets of guided modes (Pisanello et al., 2015) and allowing up to three independent stimulation points on a 1-mm-long segment of the taper (Figure 1G). The *in vivo* application of this technology was shown in both mouse motor cortex for layer-selective stimulation of GABAergic neurons and in the striatum of awake and head-restrained mice, and optrodes were realized by placing the nanostructured optical fiber beside a linear electrodes array for extracellular recording (Pisanello F. et al., 2014). On the other hand, with respect to standard optical fibers, multipoint emitting optical fibers need a higher injection power to achieve effective optogenetic control of neural activity and the total efficiency depends on the distance between the active window and the taper tip. The integration of a linear electrodes array beside the fiber, moreover, increases the invasiveness of the device.

Very recently, a series of approaches have allowed for integrated multipoint stimulation and multipoint electrical readout of neural activity (Grosenick et al., 2015). Canales et al. (2015) have developed a set of multifunctional devices based on a polymeric technology for simultaneous drug delivery, optogenetic control and extracellular recording (see representative images in Figure 1H). Together with multiple integrated functionalities, these fibers are able to better match the brain mechanical properties by virtue of the combination of different flexible materials, including poly(etherimide), poly(phenylsulfone), polycarbonate, and cyclic olefin copolymer and conductive poly-ethylene (Canales et al., 2015). Lee et al. (2015), instead, have recently proposed a new system based on optically transparent and electrically conductive ZnO semiconductor. As schematically shown in Figure 1I, the device is composed by a matrix of ZnO waveguides coated with Parylene-C up to ITO-coated tips. This configuration allows for a strong reduction of photoelectric artifacts induced by direct electrode illumination, and thereof to spatially match light delivery stimuli and electrical readout.

It is important to highlight that the main limitation of waveguides-based multipoint stimulation devices relies on the need to tether the animal to an optical bench, for coupling with the proper light injection system. For some of these approaches, animal movement and the resulting fiber bending and stretching can potentially lead to crosstalk between the different channels, and can generate inhomogeneous light delivery, in particular in the case of multimodal waveguides (Cui et al., 2013, 2014).

### Coupling of μLED with implanted optical waveguides

Hybrid approaches are instead represented by integrated technologies to couple light emitted from μLEDs into implanted waveguides. This was achieved on a flexible polyimide ribbon cable by Schwaerzle et al. using a silicon housing to align implantable optical fibers with the light sources and subsequent fixation with UV-curable adhesive fixation (Schwaerzle et al., 2015; Figure 1J). Very recently, Kwon et al. also developed a technique to couple μLEDs with microfabricated microneedles in a wireless-driven implant for multisite and bilateral stimulation of the rat visual cortex (Kwon et al., 2015; Figure 1K). It is composed by two arrays of SU8 tapered waveguides covered by a stack of ITO/Parylene-C/Gold/Parylene-C to allow for simultaneous electrical recording of neural activity in the proximity of the light delivery site (Figure 1L). The waveguides are realized on a Polydimethylsiloxane substrate and are then aligned and coupled to the μLEDs arrays placed on a polyamide cable. The result is an integrated device that, using a capacitor-based stimulator system, can be controlled via an inductive link with up to 32 bidirectional channels. If, on one hand, these methods greatly combine the advantages of implanted μLEDs and waveguide-based approaches, their main limitation is still represented by the highly divergent radiation pattern of the light sources, which do not allow for straightforward and repeatable coupling efficiency (Schwaerzle et al., 2015).

### Future challenges: Integrated light collection-delivery systems and long term experiments

Although, the technology development of last years mainly focused on devices for stimulation or inhibition of neural activity, a crucial aspect remains the possibility to simultaneously monitor neural activity. Most of the above-described devices can integrate electrodes for extracellular recording, and some of them allow for mapping of neural activity with single or multiple light-delivery sites, whose state of the art has been recently reviewed by Grosenick et al. (2015). However, in the same way classical electrical neural stimulation excite all the cells within the stimulated region, electrical readout cannot select for specific classes of neurons. This is instead possible with genetically encoded neural activity indicators (GEAIs), such as fluorescent Ca^2+^ indicators or voltage sensitive dyes (VSD). These probes respond to a variation of neural activity by changing their fluorescence intensity and are widely adopted in microscopy techniques *in vivo* to monitor electrical activity of cortical neural circuits (Svoboda et al., 1997; Kuhn et al., 2008; Warden et al., 2014). Deep brain regions, however, are widely not accessible for microscopy and the most common technique to collect light emitted from GEAIs remains the use of large core optical fibers, and are limited to a single and relatively small volume of the neural tissue (Cui et al., 2013, 2014; Gunaydin et al., 2014). The development of new techniques to efficiently collect light from sub-cortical regions is thus essential to boost the development of viable and integrated all optical bidirectional neural interfaces. Nevertheless, it is important to highlight that electrical and optical monitoring of neural activity are complementary strategies, since some electrical signals such as local field potentials have not yet an optical counterpart.

Another important challenge is represented by the possibility of using multipoint optical and optoelectronic neural interfaces in long-term experiments in untethered animals. Concerning electrical readout of neural activity, chronic implants suffer from a high variability and limited longevity of their electrical performances, as a result of failures related to a combination of biological responses of the tissue, materials stability, and mechanical properties of the device (James et al., 2013; Prasad et al., 2014). The biological aspects include (but are not limited to) damage to the blood brain barrier, inflammation responses and increased astroglial activity (Kozai et al., 2010, 2012, 2014; Saxena et al., 2013). Material failures are known to include corrosion, cracking and degradation of the insulating layer (Abhishek et al., 2012; Gilgunn et al., 2013; Prasad et al., 2014). From the point of view of the mechanical properties of the implant, most of the technologies targeting deep brain regions are based on hard materials that do not match with the softness of the brain, and therefore can hardly follow its natural movements (Hyunjung et al., 2005; Jeyakumar et al., 2005). This has increased the demand for new approaches based on flexible optoelectronic and conductive polymers (Jeong et al., 2015b), able to bend and flex to take into account pulsations and volume changes of the tissue over time [an example are the low bending stiffness multifunctional fibers described in Canales et al. (2015)]. Although very recent reports show that also the mechanical mismatch within a device can cause failures (Kozai et al., 2015), a straightforward integration between planar technologies for optical neural interfaces and flexible electronics (Kim et al., 2013; McCall et al., 2013; Goßler et al., 2014; Jeong et al., 2015a), or conceptually new approaches as syringe-injectable flexible devices (Liu et al., 2015) are therefore needed to create multifunctional devices that can match with the mechanical properties of the brain. Moreover, devices for untethered animals experiments would greatly benefit of wireless communication systems to trigger the spatiotemporal configuration of light stimuli and to retransmit the recorded data. Some approaches based on radiofrequency links (Kim et al., 2013) or optical wireless communication (Jeong et al., 2015a) have already been proposed, but next generation will have to focus on integrated systems for duplex communication allowing also for wireless and real-time optical and electrical readout of neural activity.

### Nanoparticles for optical modulation and/or readout of neural activity

Together with the new technological frontiers opened by nanotechnologies for multisite light delivery, there is a widespread agreement that the quantum properties of nano-sized materials, together with their small size and the high surface to volume ratio, can be employed to investigate alternative strategies for building next generation of optical neural interfaces. This is the case of colloidal nanoparticles, extremely small structures produced via wet-chemistry which exploit their ultra-small size to enhance quantum effects (Pellegrino et al., 2005; Carbone and Cozzoli, 2010). In particular, the reduced size often results in an enhanced sensitivity on the electromagnetic properties of the surrounding environment, and this specific feature has been exploited in two very recent works to stimulate neural activity by using plasmonic gold nanoparticles (João et al., 2015) and to evaluate the possibility of optically monitoring neural activity exploiting charge carriers dynamic in semiconductor nanocrystals (Marshall and Schnitzer, 2013).

**Figure 2:**
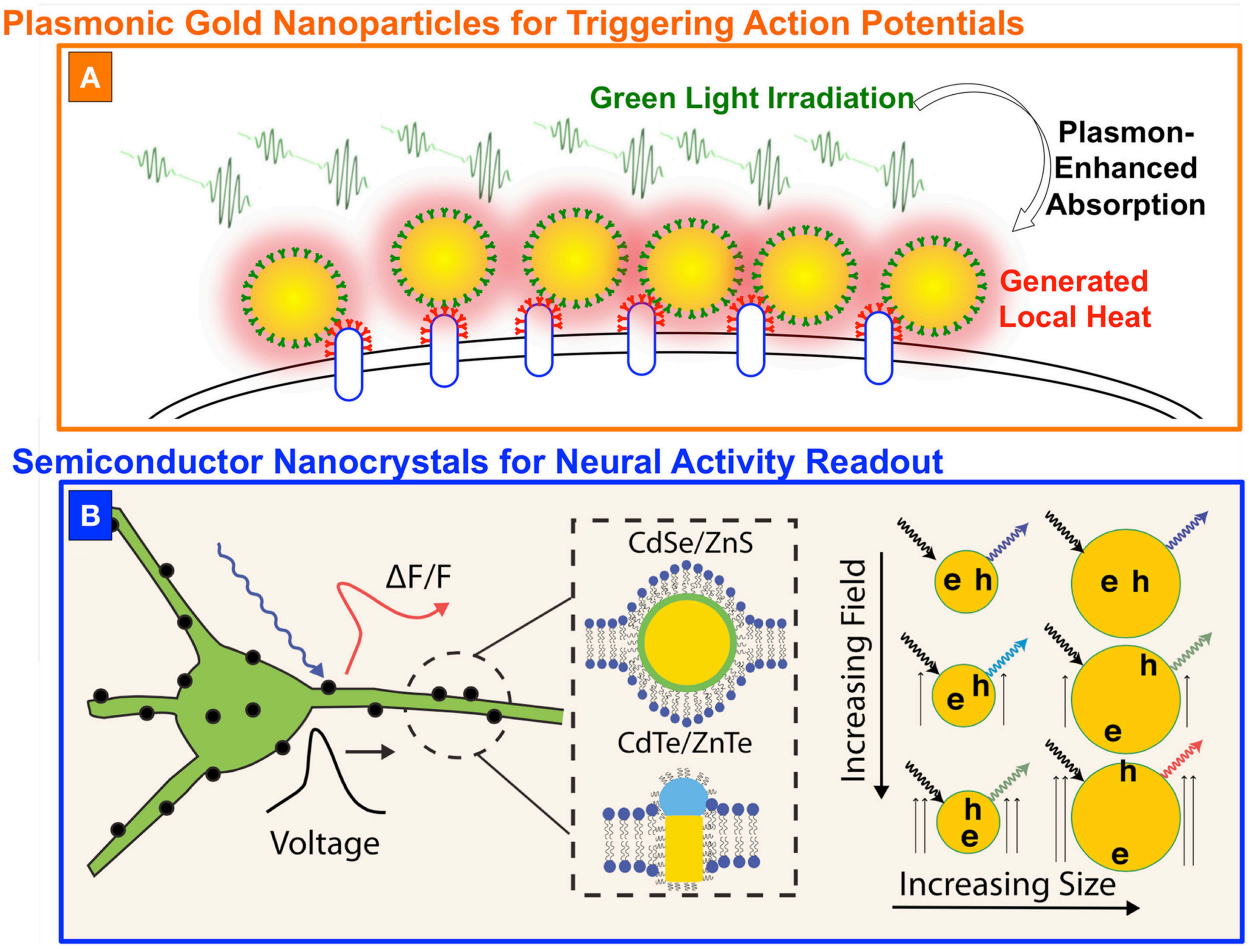
**Nanoparticles for optical modulation and/or readout of neural activity. (A)** Gold nanoparticles functionalized on the cell membrane to generate local heating (represented by the red shadow) upon green light absorption (represented by the green waves). **(B)** Semiconductor nanoscristals placed in the lipid bilayer could be used to sense voltage by detecting fluorescence fluctuations (ΔF/F) generated by the time-dependent electric field. **(B)** is reproduced with permission from Marshall and Schnitzer (2013).

In the case of plasmonic gold nanoparticles (PGNPs), the authors of João et al. (2015) suggested the use of a plasmon-mediated high absorption at green wavelengths to generate a localized heating and therefore to trigger action potentials, as schematically represented in Figure 2A. Carvalho-de-Souza et al. developed a technique to functionalize the surface of the neurons with spherical gold nanoparticles of about 20 nm in diameter, having a maximum plasmonic absorption band at 523 nm. When irradiated by green light, the particles convert the absorbed energy in local heating, inducing a variation in the membrane capacitance (Shapiro et al., 2012), rather than acting on temperature-dependent ion channels (Stanley et al., 2012), and a subsequent fast cell depolarization. If threshold potential is reached, voltage-gated channels open and the action potential is triggered (João et al., 2015). The functionalization is obtained with primary antibodies targeting different proteins on the cell membrane and secondary antibodies conjugated on the particle surface, but also exploiting a synthetic version of the Ts1 neurotoxin, which selectively binds to voltage-gated sodium channels. This latter was tested in both dorsal root ganglion neuronal cultures and hippocampal brain slices, suggesting the generality of the approach. Although, this technique does not need any genetic treatment of the tissues of interest and represents an important complement to existing technologies, it still has important challenges to face, in particular if compared to optogenetics. Indeed, one of the main peculiarities of optogenetics is the possibility to inhibit neural activity, while PGNPs can be used only for stimulation and, moreover, the particles are allowed to last into the organisms for a fixed period of time, reasonably much shorter than the almost permanent expressions of light-gated ion channels (João et al., 2015).

Another promising, but still only theoretical, application of colloidal nanoparticles is the readout of neural activity by using semiconductor nanocrystals (NCs). NCs are nanometer-sized semiconductors that present quantized levels in both valence and conduction band, rather than the quasi-continuum of states allowed in bulk semiconductors. A photon absorbed by a NC generates a bound electron-hole (e-h) pair, which can recombine following radiative (e.g., emitting a photon at lower energy) or non-radiative channels. For NCs made of II-VI semiconductors, emitted light is in the visible spectral range and, at physiological conditions, it has a well-defined single-peak emission spectrum with a ~30 nm-large Gaussian distribution around the peak wavelength. When specific types of nanoparticle are inserted into an electric field, however, the e-h pairs polarize along the direction of the applied field, and both the emission wavelength and the lifetime of the excited state change accordingly (Galland et al., 2011). Marshall et al. suggested to exploit this feature to optically read out action potentials (Marshall and Schnitzer, 2013). The model system relies in a NC, either spherical or elongated, placed into the lipid bilayer in order to have the particle sensitive to the highest possible voltage variation across the cell membrane (Figure 2B). When the membrane potential changes, the radiative lifetime changes as well, and the fraction of excitation events leading to non-radiative recombination is modified accordingly. This directly leads also to a variation of the fluorescence intensity and, overall, to the possibility to sense action potential by monitoring the fluorescence intensity of the NCs in a way very close to standard approaches used for voltage sensitive dyes. This technique have, potentially, strong advantages if compared to the use of other fluorescent indicators of neural activity, which are still facing the challenge of a limited signal to noise ratio and pronounced photobleaching. On the contrary, NCs have a strong absorption cross-sections at both one and two photons, high emission intensity, and can support longer excitation times before bleaching, making them ideal from the point of view of fluorescence stability (Spinicelli et al., 2009; Galland et al., 2011; Pisanello et al., 2013). However this remains a theoretical proposal and its implementation is mainly limited by the absence of techniques to viably and stably localize semiconductor NCs across the cell membrane of neurons.

## Conclusions and perspectives

Nanotechnology, exploiting surface and bulk nano and micromachining and self-organized nanochemistry, has dramatically improved the capability to produce devices that deliver, shape, and collect light with high spatial and temporal resolution. In the past decade all those technologies have been applied to ICT applications and for biosensing assays, with many materials and methods derived from the telecom and nanoelectronic industry.

Since the advent of different *in vivo* biophotonic methods, such as optogenetics, and new nanobiosensing approaches and clinical applications, there has been an increasing need of optical tools and convergence of integrated nanophotonic technologies toward lifescience. However, biological tissue are dispersive media and they do not allow a straightforward propagation and control of light inside organs and, specifically, in the brain.

In this paper we have shown how the scientific community is facing this challenge. So far light delivery to deep brain regions cannot be achieved without being extremely close or in contact with the region of interest, and the proposed approaches always rely on waveguides, optical fibers or wired LEDs mounted on rigid or flexible supports. At the current stage, optogenetics allow to target genetically defined classes of neurons, while the recently developed multipoint devices add the feature of controlling closely spaced neurons belonging to the same class also in deep brain regions, complementing the high spatial resolution obtained with microscopy in the firsts cortical layers. Moreover, the integration of multipoint extracellular recording of neural activity is allowing for unprecedented spatial resolved stimulation/readout in free-moving animals (Grosenick et al., 2015) and the integrated drug delivery systems improved spatial matching of viral injection, light delivery and extracellular readout (Canales et al., 2015; Jeong et al., 2015a). Although, most of these technologies were not yet used for novel biological insights, it is clear that a new generation of approaches better able to interface with the extreme complexity and diversity of brain topology and connectivity will shortly represent a key for neuroscientists to answer long-standing questions about brain functional connectivity. However, new approaches exploiting a combination of top-down and bottom up fabrication methods, nanophotonics (nanoplasmonics, quantum optical nanoantennas, etc) and new biological and neurophysiological methods are still needed. The final target is the control and distribution of light over thousands of single neurons, or even at sub-cellular level, and their wireless or waveguide-less manipulation and monitoring.

## Author contributions

All authors listed, have made substantial, direct and intellectual contribution to the work, and approved it for publication.

### Conflict of interest statement

The authors declare that the research was conducted in the absence of any commercial or financial relationships that could be construed as a potential conflict of interest.
