# Corrigendum: Micro- and Nanotechnologies for Optical Neural Interfaces

**DOI:** 10.3389/fnins.2016.00468

**Published:** 2016-10-14

**Authors:** Ferruccio Pisanello, Leonardo Sileo, Massimo De Vittorio

**Affiliations:** ^1^Center for Biomolecular Nanotechnologies, Istituto Italiano di TecnologiaLecce, Italy; ^2^Dipartimento di Ingegneria dell'Innovazione, Università del SalentoLecce, Italy

**Keywords:** nanotechnology, optogenetics, nanoparticles, neural interfaces, optical fibers

After the publication of this paper Pisanello et al., we have noticed that there was an error in the institutional affiliations of some of the authors. In particular, Leonardo Sileo, who appeared affiliated to “*Dipartimento di Ingegneria dell'Innovazione, Università del Salento, Lecce, Italy”*, should be affiliated instead only to “*Center for Biomolecular Nanotechnologies, Istituto Italiano di Tecnologia, Lecce, Italy.”* Massimo De Vittorio, who appeared affiliated only to “*Center for Biomolecular Nanotechnologies, Istituto Italiano di Tecnologia, Lecce, Italy”* should be affiliated also to “*Dipartimento di Ingegneria dell'Innovazione, Università del Salento, Lecce, Italy.”* Correct affiliations appear below the title of this corrigendum.

## Author contributions

All authors contributed equally to this work.

### Conflict of interest statement

The authors declare that the research was conducted in the absence of any commercial or financial relationships that could be construed as a potential conflict of interest.

